# Successful Knee Replacement in a Patient With a History of Multiple Knee Surgeries: A Case Report

**DOI:** 10.7759/cureus.63355

**Published:** 2024-06-28

**Authors:** Ishiqua V Patil, Prerit Sharma, Ankur Salwan, Khizar K Khan, Gajanan Pisulkar

**Affiliations:** 1 Hospital Administration, Datta Meghe Institute of Higher Education and Research, Wardha, IND; 2 Interventional Radiology, Jawaharlal Nehru Medical College, Datta Meghe Institute of Higher Education and Research, Wardha, IND; 3 Orthopedic Surgery, Jawaharlal Nehru Medical College, Datta Meghe Institute of Higher Education and Research, Wardha, IND

**Keywords:** demonstrates, contraction, osteoarthritis, degenerative, ehlers-danlos syndrome

## Abstract

This case report describes the successful total knee arthroplasty (TKA) in a 58-year-old female with a prior history of multiple knee surgeries. The patient had three prior surgical procedures. The first surgery of the patient was a partial knee replacement, the second surgery the patient underwent was an arthroscopic meniscectomy, and the third surgery was a high tibial osteotomy (HTO) that left her with an extensive amount of scar tissue and a change in physical structure. When scar tissue develops over or close to a joint, the surrounding tissues are pulled inward by this shrinking or contraction. A joint may experience restricted movement as a result of this tightness. Stretchy and excessively flexible joints are common in people with Ehlers-Danlos syndrome. This may become an issue if you need sutures for a wound because the skin is frequently not strong enough to support them. The patient already undergone three surgeries prior but still showed signs of severe pain, swelling, and stiffness in the knee which made the patient suffer more during rest position and also made it sometimes so difficult that it affected everyday tasks. In this situation when the patient consulted the doctors, the patient was suggested to undergo TKA. TKA is the method of orthopedic surgical technique that is most consistently successful and highly effective. Patients with end-stage degenerative knee osteoarthritis might expect reliable results from this surgery. The case demonstrates the preoperative planning, surgical methods, and postoperative care needed to successfully treat a complicated patient profile. Hospital protocols were followed, and the patient’s surgery was done with proper care and hygiene.

## Introduction

A frequently received technique for treating severe osteoarthritis and other crippling knee diseases is total knee arthroplasty (TKA). TKA, however, comes with special difficulties for patients who have had several knee surgeries in the past [1]. Although TKA results in amazing improvements in the patient's quality of life and exceptional success rates, there are particular difficulties while treating patients who had previous knee procedures as they already have undergone many surgeries due to which the patient has weak soft tissues, unstable joints, and abnormal anatomical landmarks, leading to difficulty in the recovery period. The patient was suggested to undergo TKA after careful observation and investigations.

This case shows how important it is to manage a difficult knee arthroplasty situation using a multidisciplinary approach in a 58-year-old female patient who had three previous knee surgeries named partial knee replacement, arthroscopic meniscectomy, and high tibial osteotomy (HTO) [2]. Due to the complexity of her surgical background, a successful outcome required careful preoperative planning, creative surgical methods, and extensive postoperative care. By discussing this case, I want to comprehend the complex process of performing TKA on patients who have had multiple procedures in the past, we have made an attempt to highlight the essence of the complicated process of performing TKA on patients with multiple previous knee surgeries [3].

## Case presentation

A 58-year-old female patient was referred to Acharya Vinoba Bhave Rural Hospital, Wardha. The patient came with complaints of severe pain, swelling, and stiffness in the knee which affected and restricted the movements in patients. Upon examination, the patient's vitals were normal. Upon asking the past history, it was noted that in 20 years, she had three knee surgeries in her past. The patient's knee pain continued despite the treatments and surgeries during previous years, and she complained that it had gotten worse over time and wanted relief. The patient explained that she had a meniscal tear which led to her first surgery known as arthroscopic meniscectomy. Years later, she had an HTO to relieve worsening osteoarthritis and fix a varus deformity. Her chronic discomfort and localized cartilage deterioration were treated with a partial knee replacement five years before the current presentation. Upon physical examination and investigations, there was a notable varus deformity, a large joint effusion, and a well-healed but evident surgical scar over the anterior knee. There was noticeable crepitus with movement and a severely limited range of motion, from 0 to 90°.

In her scans, the right knee showed signs of advanced tricompartmental osteoarthritis, including subchondral sclerosis, osteophyte production, and narrowing of the joint space. The scans also revealed indications of a prior HTO and a partial knee prosthesis, among other surgical modifications. Preoperative workup involved extensive imaging tests, MRI and X-rays, to evaluate soft tissue integrity, surrounding bone health, and osteoarthritis severity. A radiologist, a physical therapist, and an orthopedic surgeon with expertise in complex knee reconstructions worked together as a team to evaluate the patient and decide on the most suitable surgical and rehabilitative plan. The patient was given general anesthesia during surgery to put the patient in an unconscious state. Using a longitudinal midline incision, the prior surgical scar was carefully included in the surgical technique. After the partial knee prosthesis was taken out, a thorough debridement procedure was done to remove all fibrotic tissue and provide a clean surgical area. Standard bone incisions and sample components were used to ensure correct positioning and ligament balance. To guarantee smooth articulation, the patella was resurfaced, and the last implants were cemented into position. For a speedy and healthy recovery of the patient, early mobilization, careful postoperative monitoring, and effective pain management are also important.

**Figure 1:**
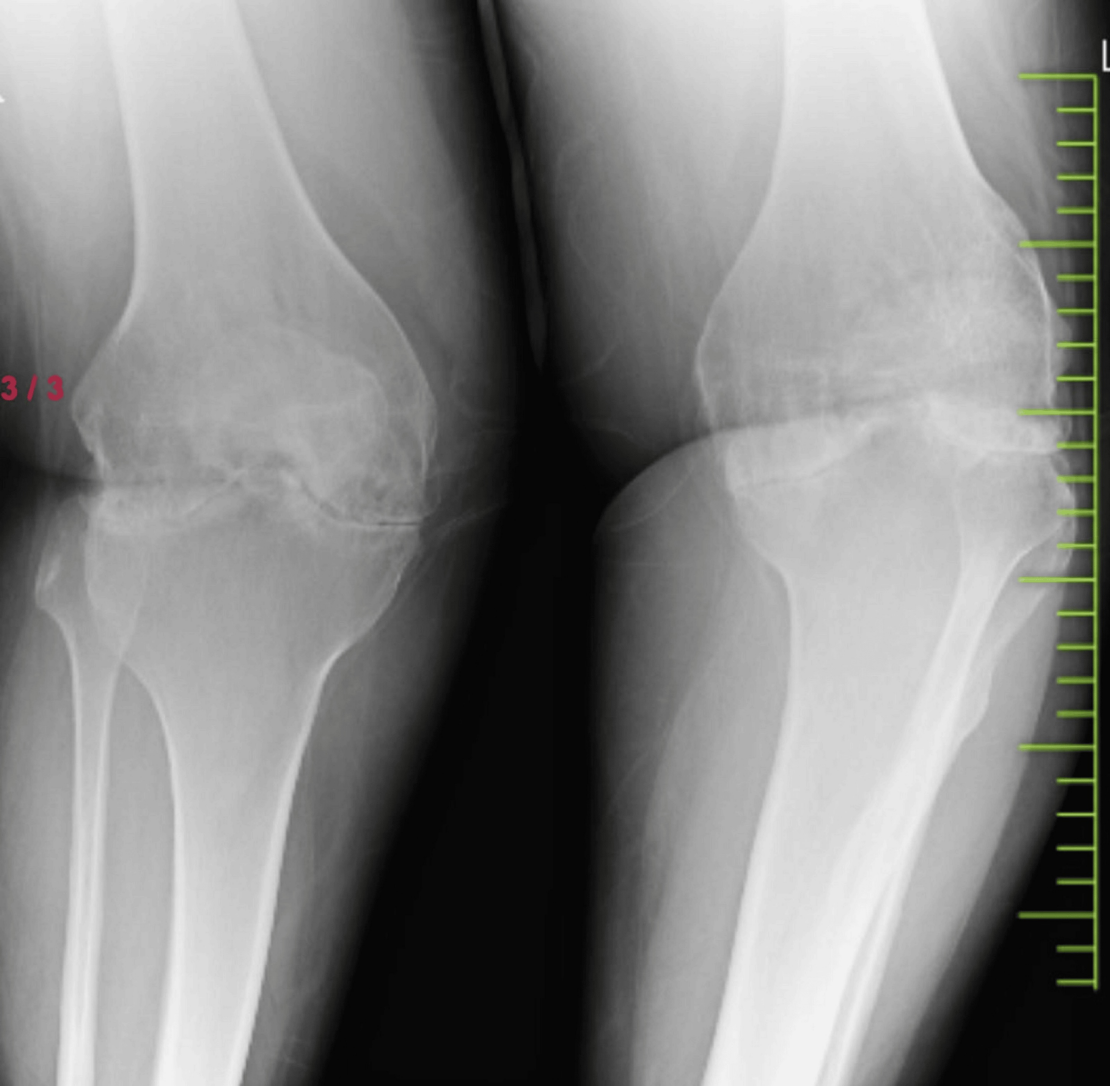
Osteoarthritis in the knee

**Figure 2:**
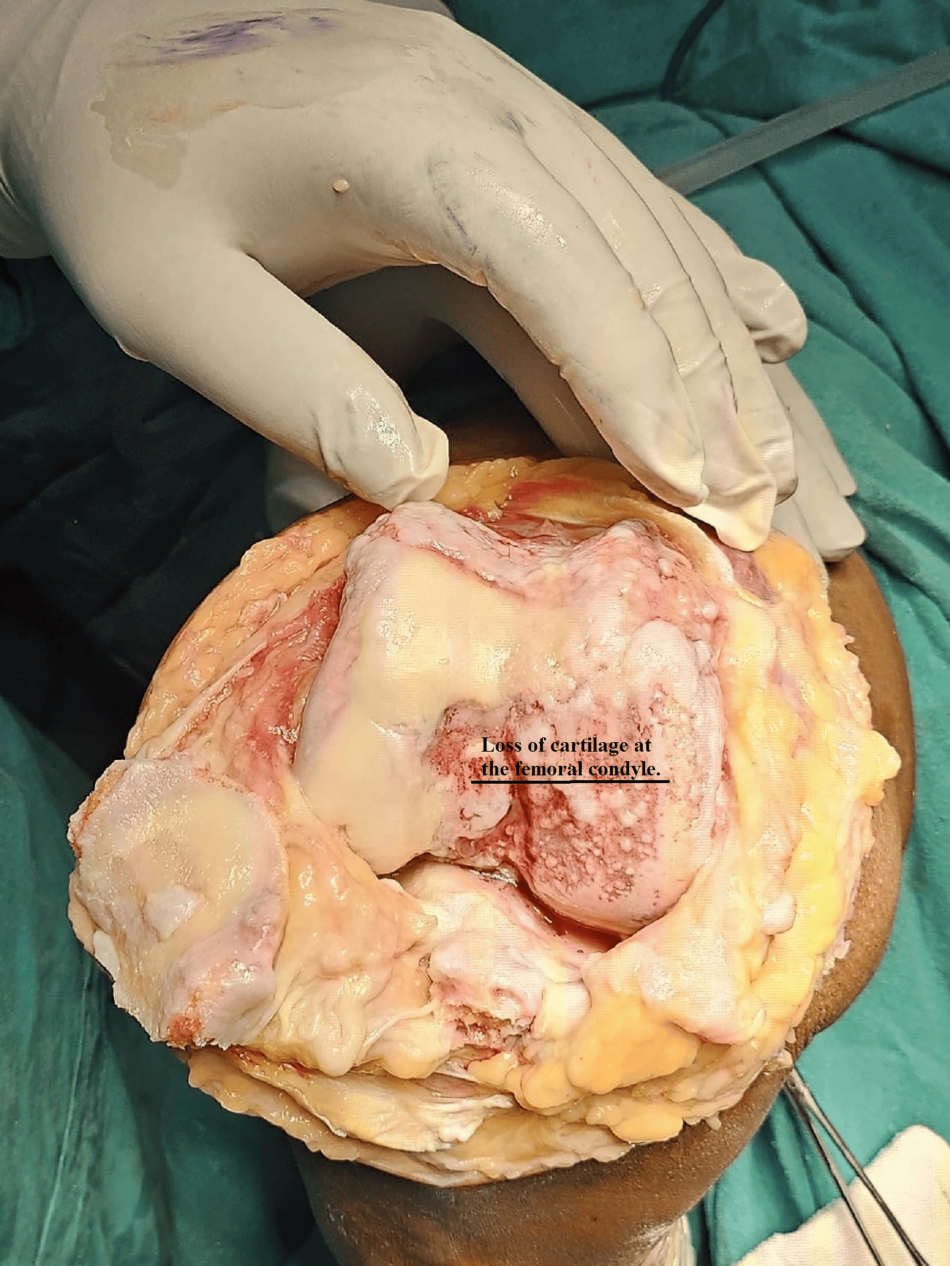
The patient's area of defect in the knee which shows loss of cartilage at the femoral condyle

**Figure 3:**
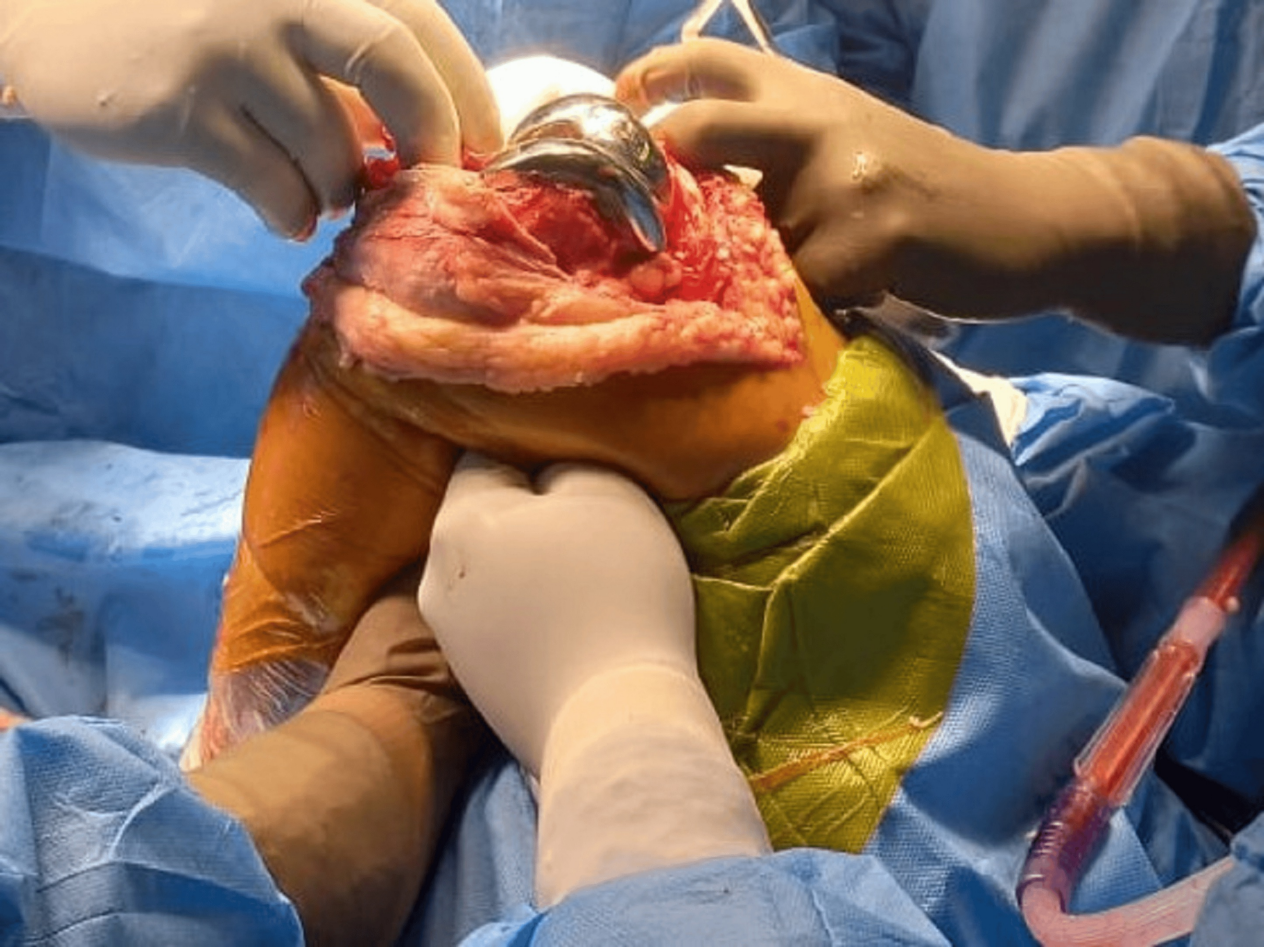
The figure shows the placement of prosthetics

**Figure 4:**
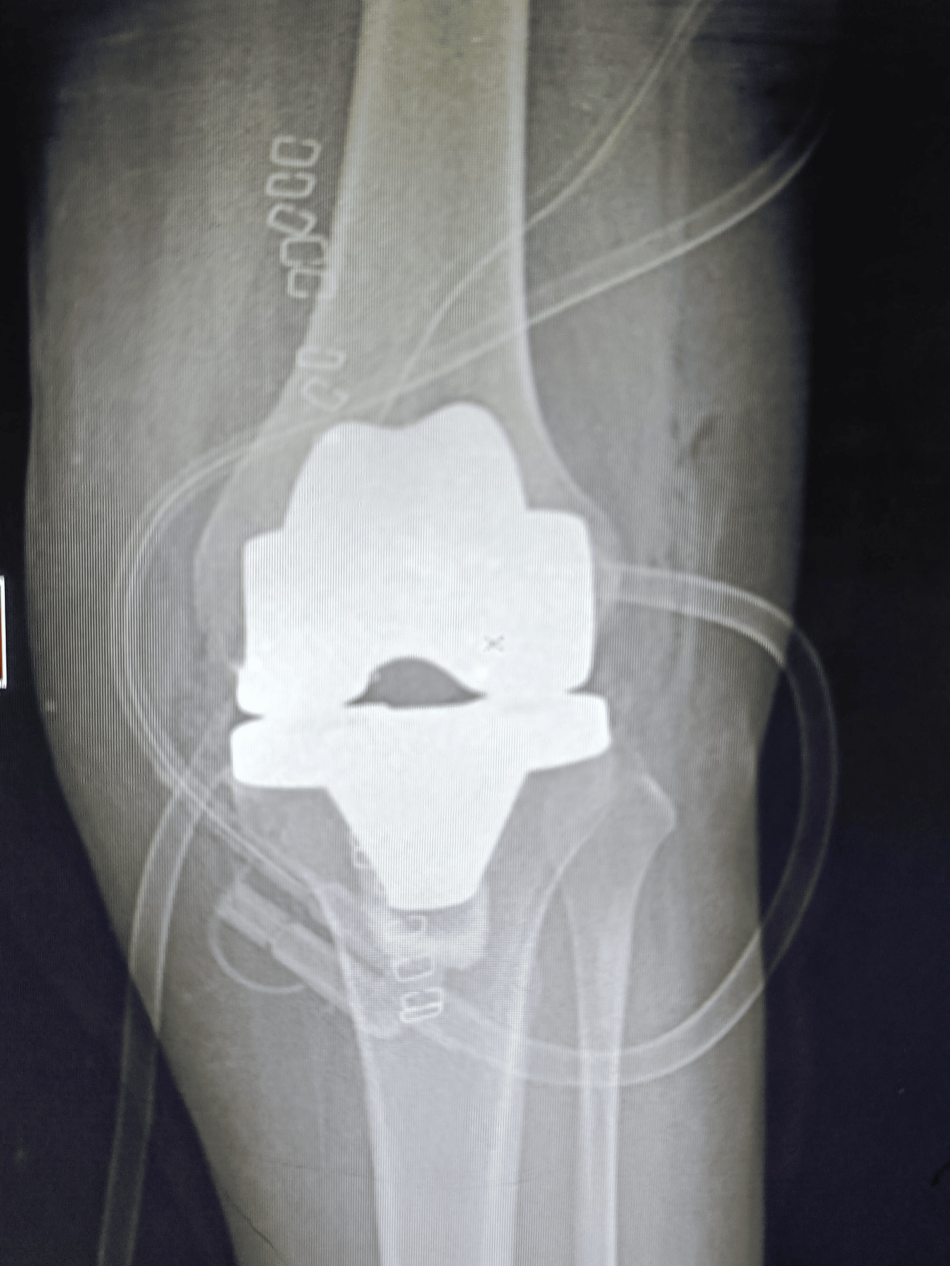
The figure shows the X-ray scan of the patient that was taken after successful surgery

Prophylactic antibiotics were given to the patient. The surgical site was constantly observed so that the patient should not get infected. The patient's recovery after surgery went smoothly and overall movement, pain level, and mobilization were improved. The patient had a range of motion from 0 to 110° at the six-week follow-up with reduced pain and no signs of an infection or other problems. Radiographs and scans showed that the prosthetic parts were positioned and aligned correctly. There were no signs of prosthesis wear or loosening, the knee joint was stable, and on re-examination, she was not having any kind of pain and discomfort. At the six-month follow-up, the patient reported a significant improvement in her quality of life. Figure 1 shows the preoperative condition of the patient through an X-ray which shows osteoarthritis in the patient. Figure 2 shows the patient's area of defect in the knee which shows loss of cartilage at the femoral condyle. Figure 3 shows the placement of prosthetics. Figure 4 shows the X-ray scan of the patient that was taken after successful surgery.

The patient treatment consisted of basic evaluation of patient's health with the help of detailed medical history, physical examination, and scans MRI, CT, and X-ray; this report helped doctors understand the depth of the patient's case and accordingly planned operation. The patient was advised to undergo surgery for good results and permanent relief from prolonged pain and discomfort. The patient was informed about the procedure, risks, and postoperative care. A signed consent form was obtained from the patient before undergoing surgical procedure. The patient underwent TKA under general anesthesia with a regional nerve block, and a major focus was made on managing scar tissue as well as selecting appropriate prosthetics based on the scans and intraoperative findings. Sterile techniques and tranexamic acid were used to reduce bleeding. Overall, a proper infection and blood management care was taken. After the surgery, the patient was kept under observation; thus, the postoperative care consisted of employing multimodal analgesia (opioids, nonsteroidal anti-inflammatory drugs (NSAIDs), acetaminophen, nerve blocks) for pain management.

The postoperative treatment consisted of early mobilization which was done within 24 hours with the use of assistive devices for support. Regular monitoring and observation were made to avoid unnecessary infection, and a regular dressing was done. The patient was educated about the importance of physical therapy and was suggested to properly follow a diet chart given by the dietitian for a healthy and speedy recovery. The patient reported improvement in the knee function on a six-month follow-up with no signs of infection and significant reduction in pain allowing proper movement and mobilization to the patient. The X-ray reports showed that the patient's knee attained a good range of motion with stability, and the prosthetic components were also properly aligned and integrated. The patient was advised to continue follow-up regularly to keep tract on the knee replacement functionality.

## Discussion

TKA, known as knee replacement surgery, is a surgical procedure for operating patients with severe knee joint degeneration, pain reduction, and function restoration. However, there are many obstacles and difficulties when performing TKA on patients who have had several knee surgeries in the past. This case study explains a successful knee replacement performed on a patient with a complicated prior surgical history and was a challenge and a responsibility for surgeons to perform it safely so that the patient does not suffer after surgery, unlike his past experiences. Thus, the preoperative and postoperative considerations were equally important from both the patient's and the doctors' perspectives as this will contribute to the success of the treatment [4]. Patients who have had several knee surgeries in the past most often show changes in soft tissue, scarring, and altered anatomy which make the prosthetic installation and surgical technique more difficult [5]. The study examines the outcomes of TKA in patients who had previous knee surgeries, concluding that TKA provides significant pain relief and improved function despite the complexity added by prior surgeries [6].

Preoperative preparation included detailed imaging examinations, such as MRIs and X-rays, to evaluate the condition of the soft tissues and ligaments, the extent of prior surgical operations, and the condition of the residual bone stock in the patient. Due to the patient's several surgeries in past, the chances and possibility for infection were higher [7]. Doctors discussed, and a specific surgical strategy was planned with the help of meticulous examination of previous surgical information. Considering the long surgical history of the patient, good care was taken to reduce intraoperative difficulties. Using an existing incision line was expected to reduce the possibility of complications throughout the healing process and reduce scarring. It was important to do significant adhesiolysis intraoperatively to remove dense scar tissue from the knee joint in order to increase mobility. It was important to remove the old hardware carefully to prevent the bone damage [8]. Various factors influence TKA outcomes, including prior knee surgeries of the patient. It supports the idea that TKA remains effective even in complex cases with previous surgeries [9].

Specialized equipment and techniques were used to regulate the affected anatomy and place the prosthetic components accordingly. The best possible alignment and fit of the prosthesis was made possible by intraoperative navigation and specially designed guides based on preoperative imaging [10]. This high-risk patient's postoperative treatment was planned with their higher risk of complications in view. The use of long-term antibiotics was one of the strict infection control measures implemented. The patient was observed and monitored constantly so that he should not get any infection. Regular and timely blood tests and imaging scans were done to detect if any issues arises [11]. The major focus was on the rehabilitation process of the patient for which included early mobilization, to avoid stiffness and to increase the range of motion. Physiotherapy was scheduled for improvement of joints and movements to encourage a speedy recovery [12]. This case study evaluates the impact of prior knee surgeries on the outcomes of TKA.

## Conclusions

This case report shows that a successful knee replacement is possible even for patients who have had many previous knee surgeries. Careful planning and surgical intervention using proper implants and regular follow-up can lead to good outcomes, helping patients regain mobility and reduce pain. The patient was satisfied with the treatment and showed a healthy recovery with good progress. 
